# Associations of systemic immune-inflammation index and aggregate index of systemic inflammation with acute gouty arthritis in men: a cross-sectional study

**DOI:** 10.3389/fmed.2026.1713446

**Published:** 2026-05-14

**Authors:** Ziran Xiu, Zhengnan Gao, Lan Luo, Peiyang Yu

**Affiliations:** 1 Clinical Skills Training Center, Central Hospital of Dalian University of Technology (Dalian Municipal Central Hospital), Dalian, Liaoning, China; 2 Endocrinology and Metabolism, Central Hospital of Dalian University of Technology (Dalian Municipal Central Hospital), Dalian, Liaoning, China; 3 Department of Urology, Central Hospital of Dalian University of Technology (Dalian Municipal Central Hospital), Dalian, Liaoning, China

**Keywords:** acute gouty arthritis, AISI, complete blood cell count-derived, cross-sectional study, SII

## Abstract

**Objective:**

This study aims to evaluate the associations of complete blood cell count-derived inflammatory markers—including monocyte-to-lymphocyte ratio (MLR), neutrophil-to-lymphocyte ratio (NLR), platelet-to-lymphocyte ratio (PLR), Systemic Immune-Inflammation Index (SII), systemic inflammatory response index (SIRI), and aggregate index of systemic inflammation (AISI)—with acute gouty arthritis in males.

**Methods:**

A cross-sectional study was conducted in 380 males from the Department of Endocrinology and Department of Physical Examination, Central Hospital of Dalian University of Technology, between January 2022 and January 2024. Multivariable logistic regression models were used to investigate the independent associations between six inflammatory markers and acute gouty arthritis. Restricted cubic splines (RCS) were employed to model the dose–response relationships of inflammatory markers with acute gouty arthritis. Subgroup analyses were performed to identify susceptible populations. The diagnostic capabilities of the inflammatory markers were evaluated and compared using receiver operating characteristic (ROC) curves.

**Results:**

A total of 380 male participants were included, with a mean age of 54 years. Among them, 108 participants had AGA, giving a prevalence of 28.4%. Significant associations with AGA were observed for monocyte-to-lymphocyte ratio (MLR), neutrophil-to-lymphocyte ratio (NLR), platelet-to-lymphocyte ratio (PLR), Systemic Immune-Inflammation Index (SII), systemic inflammatory response index (SIRI), and aggregate index of systemic inflammation (AISI). Further analysis using RCS revealed nonlinear dose–response relationships between SII and AGA (*p*-nonlinear = 0.001), as well as between AISI and AGA (*p*-nonlinear <0.001). Subgroup analysis showed that inflammatory markers (NLR, PLR, SII, SIRI, and AISI) were more effective in assessing AGA onset among men with fatty liver. ROC analysis indicated that when compared with other inflammatory markers (MLR, NLR, PLR, and SIRI), SII and AISI demonstrated superior diagnostic accuracy and discriminatory power in assessing the risk of AGA in men.

**Conclusion:**

In men, AGA is closely associated with inflammatory markers. In addition, compared with other inflammatory markers (MLR, NLR, PLR, and SIRI), SII and AISI may serve as more accurate indicators for the diagnosis of AGA.

## Introduction

Gout affects individuals worldwide, with a rising prevalence among younger populations (1). Acute gouty arthritis (AGA) is an inflammatory disorder defined by hyperuricemia and the deposition of monosodium urate (MSU) crystals in joints and tissues (2). The acute phase is typically characterized by an abrupt onset, manifesting as severe joint pain, cutaneous erythema, elevated skin temperature, and joint swelling. Recurrent acute gout attacks may progress to chronic gouty arthritis and tophus formation after years (2, 3). Additionally, individuals with gout are at an elevated risk of cardiovascular disease and chronic kidney disease (1). Thus, early intervention is essential to halt the progression of AGA.

Inflammatory markers are established biomarkers for evaluating disease severity and guiding therapeutic strategies in patients with AGA. Many inflammatory factors, such as IL-1β, IL-6, TNF-α, and other cytokines, are involved in the inflammatory response induced by urate crystals and are crucial in the inflammatory cascade amplification response (4). However, given the high cost of detecting inflammatory factors, they are rarely used as routine clinical assay indices. Erythrocyte sedimentation rate (ESR) and C-reactive protein (CRP) are often used as markers of acute gout in the clinical context, but these two inflammatory markers are nonspecific in gout and have limited ability to assess gout disease activity. Therefore, there is a need to identify the inflammatory markers associated with disease activity. The complete blood cell count-derived is a new nonspecific inflammatory index that includes the monocyte-to-lymphocyte ratio (MLR), neutrophil-to-lymphocyte ratio (NLR), platelet-to-lymphocyte ratio (PLR), Systemic Immune-Inflammation Index (SII), systemic inflammatory response index (SIRI), and aggregate index of systemic inflammation (AISI). These indicators better reflect systemic inflammation, are readily available and inexpensive, and have been used to assess the degree of inflammation and outcomes in rheumatoid arthritis, ankylosing spondylitis, and systemic lupus erythematosus (5–9). Previous studies have investigated the relationship between blood cell-derived inflammatory markers and acute gouty arthritis (10), but it is not comprehensive. In addition, there is still significant debate about the advantages and limitations of complete blood cell count -derived inflammatory markers, and the relationship between inflammatory markers and acute gouty arthritis has not been comprehensively evaluated.

Thus, the present study aimed to further explore these associations and comprehensively evaluate the potential of these six blood cell-derived inflammatory markers for assessing acute gout flares. Given the higher prevalence of gout in men (11), the study population was restricted to male participants. The objective was to develop a more accurate assessment tool to facilitate the early detection and prevention of gout.

## Materials and methods

### Study population

**Figure 1:**
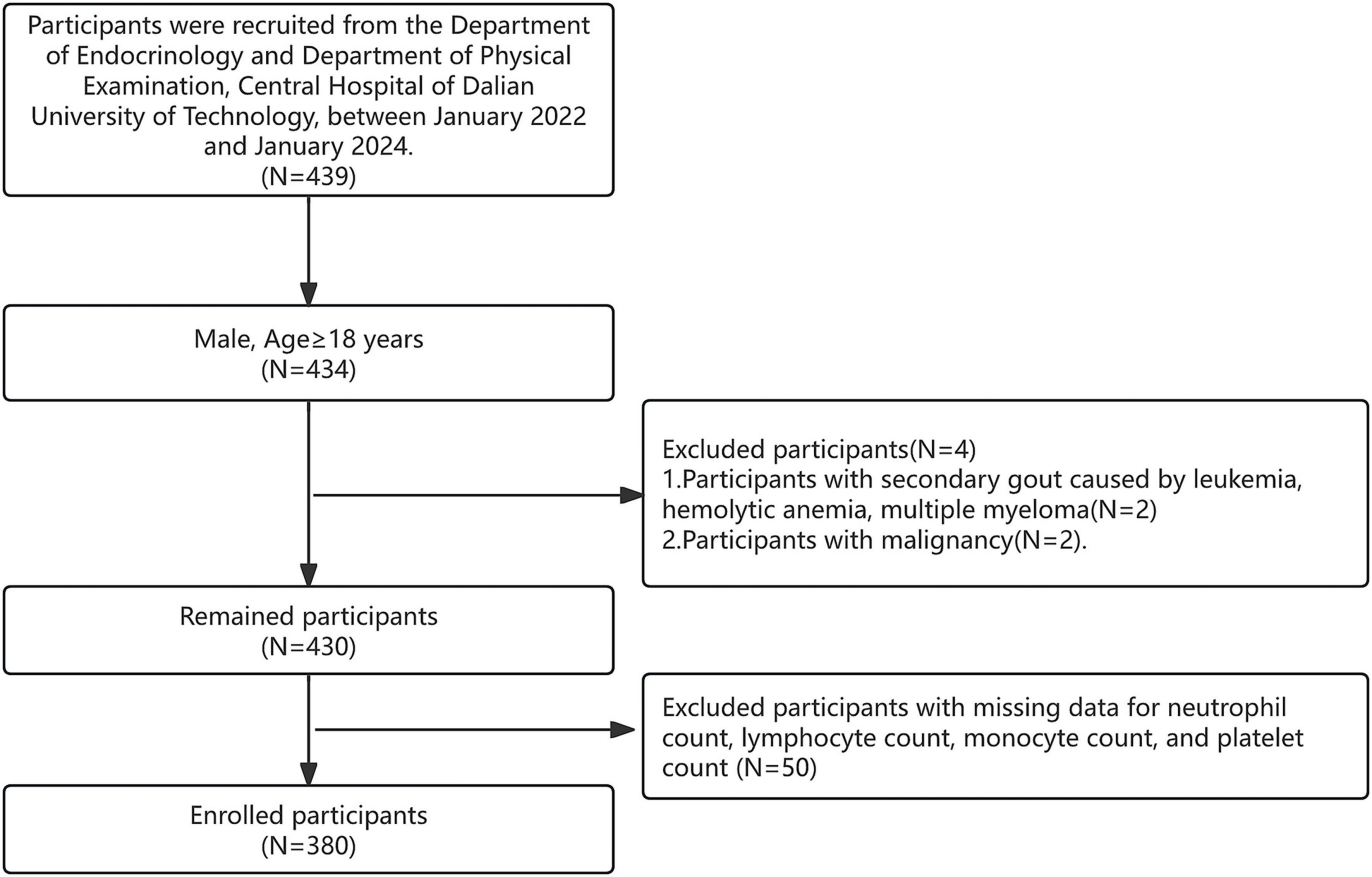
Selection flowchart.

This study included 439 patients from the Department of Endocrinology and Department of Physical Examination, Central Hospital of Dalian University of Technology, between January 2022 and January 2024. Of these, 434 male participants aged ≥18 years were selected. Exclusion criteria were as follows: 2 patients with secondary gout caused by leukemia, hemolytic anemia, or multiple myeloma; and 2 patients with malignant tumors. Additionally, 50 participants with incomplete data on neutrophil, lymphocyte, monocyte, and platelet counts were excluded. Ultimately, 380 participants were included in this cohort study. Of these, 108 individuals were diagnosed with acute gouty arthritis (AGA) based on the 2015 ACR/EULAR gout classification criteria (12) and allocated to the AGA group. Synovial fluid aspiration for monosodium urate (MSU) crystal detection was not performed due to clinical feasibility constraints. Meanwhile, 272 healthy participants who underwent physical examinations at the Department of Physical Examination were assigned to the control group, as shown in Figure 1. All participants provided written informed consent, and the protocol was approved by the Ethical Review Committee of Central Hospital of Dalian University of Technology (Dalian Municipal Central Hospital) (2023-057-29).

### Calculation of inflammatory indices

MLR was calculated with the formula: monocyte count (10^9^/L)/lymphocyte count (10^9^/L).

NLR was calculated with the formula: neutrophil counts (10^9^/L)/lymphocyte counts (10^9^/L).

PLR was calculated with the formula: platelet count (10^9^/L)/lymphocyte count (10^9^/L).

SII was calculated with the formula: neutrophil counts (10^9^/L) × platelet counts (10^9^/L)/lymphocyte counts (10^9^/L).

SIRI was calculated with the formula: neutrophil counts (10^9^/L) × monocyte counts (10^9^/L)/lymphocyte counts (10^9^/L).

AISI was calculated with the formula: [neutrophil count (10^9^/L) × platelet count (10^9^/L) × monocyte count (10^9^/L)]/lymphocyte count (10^9^/L).

### Covariates

Information on participants’ baseline data was collected via questionnaires and laboratory tests, including age, sex, smoking status, alcohol consumption, and histories of hypertension, diabetes, hyperlipidemia, kidney stones, and fatty liver, as well as body mass index (BMI). Laboratory measurements comprised total cholesterol (TC), triglycerides (TG), high-density lipoprotein cholesterol (HDL-C), low-density lipoprotein cholesterol (LDL-C), glucose (Glu), serum creatinine (SCr), serum uric acid (SUA), aspartate aminotransferase (AST), alanine aminotransferase (ALT), white blood cell count (WBC), neutrophil count (NEU), lymphocyte count (LYM), monocyte count (MONO), and platelet count (PLT).

### Statistical analysis

Normality tests were conducted for all data. Variables that did not conform to a normal distribution were analyzed using the Mann–Whitney *U*-test. Results were reported as median values with interquartile ranges (Q25, Q75). Categorical variables were described by frequency and percentage (*n*%), and group differences were assessed via the chi-square test. All inflammatory markers were standardized via the *Z*-score method. Multifactorial logistic regression was then employed to examine the association between inflammatory markers and AGA. In the absence of predefined cut-off points, the tertiles method was applied for analysis. Three analytical models were constructed: Model 1 (unadjusted), Model 2 (adjusted for age), and Model 3 (adjusted for multiple variables, including age, TG, LDL-C, SCr, ALT, alcohol consumption, hypertension, kidney stones, and fatty liver). Trend tests for ordered categorical variables were performed using the Cochran-Armitage trend test. Dose–response relationships between inflammatory markers and AGA were evaluated via restricted cubic splines (RCS). Subgroup analyses were conducted to investigate whether covariates (e.g., age, alcohol consumption, hypertension, kidney stones, and fatty liver) modified the association between inflammatory markers and AGA, with interactions defined as significant at *p* < 0.05. The diagnostic performance of inflammatory markers for AGA was assessed using ROC curve analysis, with area under the curve (AUC), sensitivity, and specificity reported. All statistical analyses were performed using EmpowerStats (version 4.2) and R software (version 4.3.2). A two-sided *p*-value<0.05 was considered statistically significant.

## Results

**Figure 2:**
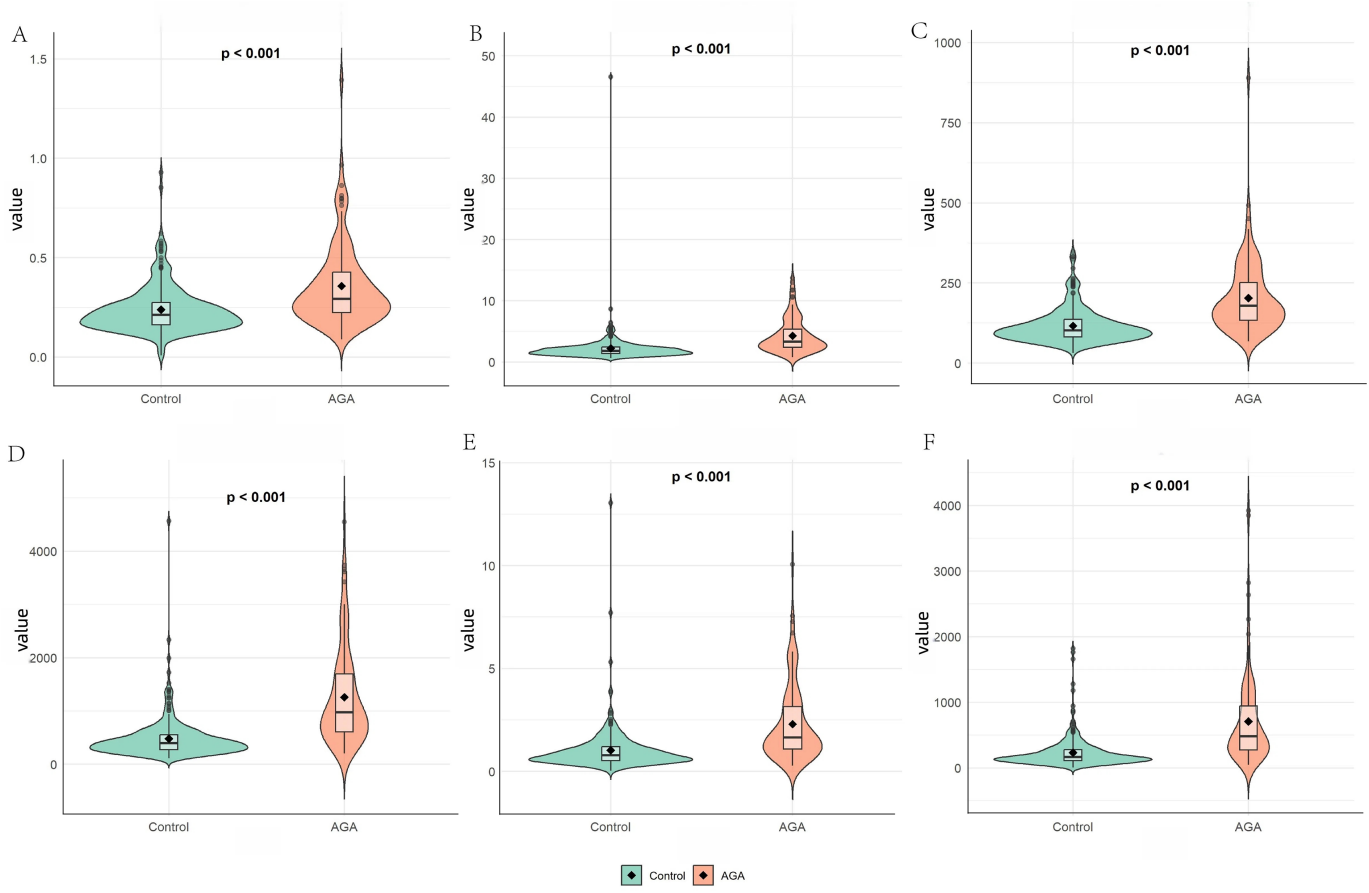
Comparison of inflammatory markers between the AGA group and control group: MLR **(A)**, NLR **(B)**, PLR **(C)**, SII **(D)**, SIRI **(E)**, and AISI **(F)**. MLR, monocyte-to-lymphocyte ratio; NLR, neutrophil-to-lymphocyte ratio; PLR, platelet-to-lymphocyte ratio; SII, Systemic Immune-Inflammation Index; SIRI, systemic inflammatory response index; AISI, aggregate index of systemic inflammation.

A total of 380 male patients were enrolled in this study, including 108 patients in the AGA group with a mean age of 55 years and 272 patients in the control group with a mean age of 53 years. No significant age difference was observed between the two groups (*p* > 0.05). As shown in Table 1; Figure 2, those who developed AGA had significantly higher baseline levels of LDL-C, SCr, ALT, WBC, NEU, MONO, PLT, MLR, NLR, PLR, SII, SIRI, and AISI, but significantly lower levels of TG and LYM. Additionally, the AGA group had higher baseline prevalences of drinking, hypertension, kidney stone, and fatty liver.

**Table 1:** Baseline characteristics.

Characteristics	Overall	AGA	Control	*p*-value
	(*N* = 380)	(*N* = 108)	(*N* = 272)	
Age	54 (43, 64)	55 (42, 66)	53 (43, 63)	0.430
BMI (kg/m^2^)	26.90 (24.20, 29.50)	25.90 (23.98, 28.48)	27.40 (24.80, 30.10)	0.116
TC (mmol/L)	4.48 (3.84, 5.00)	4.33 (3.75, 4.95)	4.50 (3.92, 5.04)	0.171
TG (mmol/L)	1.77 (1.22, 3.03)	1.36 (1.02, 1.79)	2.16 (1.36, 3.45)	<0.001
HDL-C (mmol/L)	0.88 (0.77, 1.01)	0.88 (0.76, 0.99)	0.88 (0.77, 1.012)	0.184
LDL-C (mmol/L)	2.73 (2.20, 3.31)	3.00 (2.36, 3.51)	2.67 (2.12, 3.16)	0.002
SCr (μmol/L)	79.00 (67.98, 93.00)	85.90 (74.25, 103.43)	75.00 (65.00, 89.00)	<0.001
SUA (μmol/L)	461.00 (374.00, 526.00)	459.00 (363.00, 550.25)	462.00 (375.50, 520.00)	0.136
Glu (mmol/L)	5.62 (5.22, 6.53)	5.59 (4.96, 6.67)	5.64 (5.29, 6.51)	0.545
ALT (U/L)	15.00 (12.00, 25.00)	27.00 (18.00, 51.00)	14.00 (11.00, 20.00)	<0.001
AST (U/L)	20.00 (17.00, 25.00)	19.00 (14.75, 28.25)	20.00 (18.00, 24.00)	0.307
WBC (×10^9^/L)	6.94 (5.67, 8.39)	7.84 (6.35, 10.19)	6.60 (5.50, 7.95)	<0.001
NEU (×10^9^/L)	4.14 (3.11, 5.39)	5.41 (4.16, 7.23)	3.79 (2.95, 4.75)	<0.001
MONO (×10^9^/L)	0.45 (0.37, 0.59)	0.51 (0.39, 0.67)	0.43 (0.36, 0.53)	<0.001
LYM (×10^9^/L)	2.00 (1.50, 2.49)	1.70 (1.22, 2.10)	2.09 (1.60, 2.50)	<0.001
PLT (×10^9^/L)	230.50 (188.75, 278.25)	278.00 (225.50, 352.25)	215.50 (178.00, 262.00)	<0.001
MLR	0.23 (0.17, 0.32)	0.29 (0.22, 0.43)	0.21 (0.16, 0.28)	<0.001
NLR	2.15 (1.49, 3.05)	3.34 (2.40, 5.34)	1.84 (1.37, 2.43)	<0.001
PLR	114.83 (87.55, 167.52)	179.31 (133.45, 251.71)	102.51 (81.76, 136.11)	<0.001
SII	469.93 (313.12, 790.75)	978.49 (608.03, 1696.20)	400.11 (277.70, 553.38)	<0.001
SIRI	0.97 (0.60, 1.55)	1.65 (1.08, 3.15)	0.78 (0.53, 1.20)	<0.001
AISI	216.80 (128.06, 396.46)	483.48 (273.76, 942.65)	164.14 (112.88, 279.17)	<0.001
Smoking, *n* (%)				0.162
Yes	193 (50.8%)	61 (56.5%)	132 (48.5%)	
No	187 (49.2%)	47 (43.5%)	140 (51.5%)	
Drinking, *n* (%)				0.006
Yes	183 (48.2%)	64 (59.3%)	119 (43.7%)	
No	197 (51.8%)	44 (40.7%)	153 (56.3%)	
Hypertension, *n* (%)				<0.001
Yes	137 (36.1%)	58 (53.7%)	79 (29%)	
No	243 (63.9%)	50 (46.3%)	193 (71%)	
Diabetes, *n* (%)				0.492
Yes	79 (20.8%)	20 (18.5%)	59 (21.7%)	
No	301 (79.2%)	88 (81.5%)	213 (78.3%)	
Hyperlipemia, *n* (%)				0.571
Yes	47 (12.4%)	15 (13.9%)	32 (11.8%)	
No	333 (87.6%)	93 (86.1%)	240 (88.2%)	
Kidney stone, *n* (%)				<0.001
Yes	16 (4.2%)	12 (11.1%)	4 (1.5%)	
No	364 (95.8%)	96 (88.9%)	268 (98.5%)	
Fatty liver, *n* (%)				0.036
Yes	95 (25%)	35 (32.4%)	60 (22.1%)	
No	285 (75%)	73 (67.6%)	212 (77.9%)	

Data are expressed as mean (P25, P75) or number (percent). BMI, body mass index; HDL-C, high-density lipoprotein cholesterol; LDL-C, low-density lipoprotein cholesterol; TC, total cholesterol; TG, triacylglycerol; SCr, serum creatinine; SUA, serum uric acid; WBC, white blood cell count; NEU, neutrophil count; LYM, lymphocyte count; MONO, monocyte count; PLT, platelet count; MLR, monocyte-to-lymphocyte ratio; NLR, neutrophil-to-lymphocyte ratio; PLR, platelet-to-lymphocyte ratio; SII, Systemic Immune-Inflammation Index; SIRI, systemic inflammatory response index; AISI, aggregate index of systemic inflammation.

Table 2 summarizes the associations between inflammatory markers and AGA. All indices were standardized using the *Z*-score method to estimate the relative risk increase per standard deviation (SD) change. Statistical significance of these associations was evaluated via score tests for linear trends. In the fully adjusted logistic regression model, NLR, MLR, PLR, SII, SIRI, and AISI were significantly positively associated with AGA (MLR: OR = 2.42, 95% CI: 1.75, 3.34; NLR: OR = 3.34, 95% CI: 2.01, 5.55; PLR: OR = 4.20, 95% CI: 2.72, 6.47; SII: OR = 6.24, 95% CI: 3.55, 10.95; SIRI: OR = 3.12, 95% CI: 2.06, 4.72; AISI: OR = 5.58, 95% CI: 3.33, 9.35). Furthermore, across tertiles of each index, the risk of AGA increased in a stepwise manner. Cochran-Armitage trend tests confirmed significant positive trends for all indices (all *p*-trend <0.001). Collectively, these findings indicate that elevated levels of NLR, MLR, PLR, SII, SIRI, and AISI are independently associated with an increased risk of AGA among males.

**Table 2:** Relationship between inflammatory markers and AGA.

Variable		Model 1		Model 2		Model 3	
		OR (95%CI)	*p*-value	OR (95%CI)	*p*-value	OR (95%CI)	*p*-value
MLR		2.17 (1.66, 2.83)	<0.001	2.19 (1.67, 2.86)	<0.001	2.42 (1.75, 3.34)	<0.001
MLR	Q1	1		1		1	
	Q2	1.96 (1.03, 3.72)	0.041	1.97 (1.03, 3.74)	0.039	1.71 (0.76, 3.83)	0.195
	Q3	5.21 (2.83, 9.57)	<0.001	5.20 (2.83, 9.56)	<0.001	5.24 (2.41, 11.41)	<0.001
*p*-trend		<0.001		<0.001		<0.001	
NLR		3.59 (2.37, 5.44)	<0.001	3.60 (2.37, 5.46)	<0.001	3.34 (2.01, 5.55)	<0.001
NLR	Q1	1		1		1.0	
	Q2	1.60 (0.77, 3.31)	0.205	1.59 (0.77, 3.29)	0.213	1.47 (0.58, 3.69)	0.416
	Q3	10.82 (5.60, 20.87)	<0.001	10.74 (5.56, 20.75)	<0.001	17.32 (6.92, 43.37)	<0.001
*p*-trend		<0.001		<0.001		<0.001	
PLR		3.75 (2.69, 5.24)	<0.001	3.75 (2.68, 5.23)	<0.001	4.20 (2.72, 6.47)	<0.001
PLR	Q1	1		1		1.0	
	Q2	2.60 (1.14, 5.93)	0.023	2.59 (1.14, 5.91)	0.024	5.55 (1.87, 16.47)	0.002
	Q3	20.69 (9.62, 44.53)	<0.001	20.59 (9.56, 44.35)	<0.001	48.78 (15.66, 151.91)	<0.001
*p*-trend		<0.001		<0.001		<0.001	
SII		5.29 (3.44, 8.13)	<0.001	5.36 (3.48, 8.25)	<0.001	6.24 (3.55, 10.95)	<0.001
SII	Q1	1		1		1.0	
	Q2	2.14 (0.70, 6.53)	0.180	2.12 (0.70, 6.47)	0.186	3.91 (0.96, 15.95)	0.058
	Q3	7.147 (2.62, 19.52)	<0.001	7.04 (2.57, 19.27)	<0.001	12.96 (3.38, 49.64)	<0.001
*p*-trend		<0.001		<0.001		<0.001	
SIRI		2.94 (2.09, 4.15)	<0.001	2.98 (2.11, 4.22)	<0.001	3.12 (2.06, 4.72)	<0.001
SIRI	Q1	1		1		1.0	
	Q2	2.72 (1.34, 5.50)	0.005	2.68 (1.32, 5.43)	0.006	2.79 (1.09, 7.13)	0.032
	Q3	9.11 (4.66, 17.83)	<0.001	9.07 (4.64, 17.76)	<0.001	18.63 (7.16, 48.48)	<0.001
*p*-trend		<0.001		<0.001		<0.001	
AISI		4.69 (3.04, 7.23)	<0.001	4.77 (3.09, 7.37)	<0.001	5.58 (3.33, 9.35)	<0.001
AISI	Q1	1		1		1.0	
	Q2	2.59 (1.21, 5.52)	0.014	2.57 (1.21, 5.50)	0.014	2.20 (0.83, 5.84)	0.113
	Q3	13.69 (6.72, 27.87)	<0.001	13.64 (6.70, 27.79)	<0.001	25.91 (9.56, 70.19)	<0.001
*p*-trend		<0.001		<0.001		<0.001	

Model 1: no adjustment.

Model 2: adjusted for age.

Model 3: adjusted for age, TG, LDL-C, SCr, ALT, drinking, hypertension, kidney stone and fatty liver.

**Figure 3:**
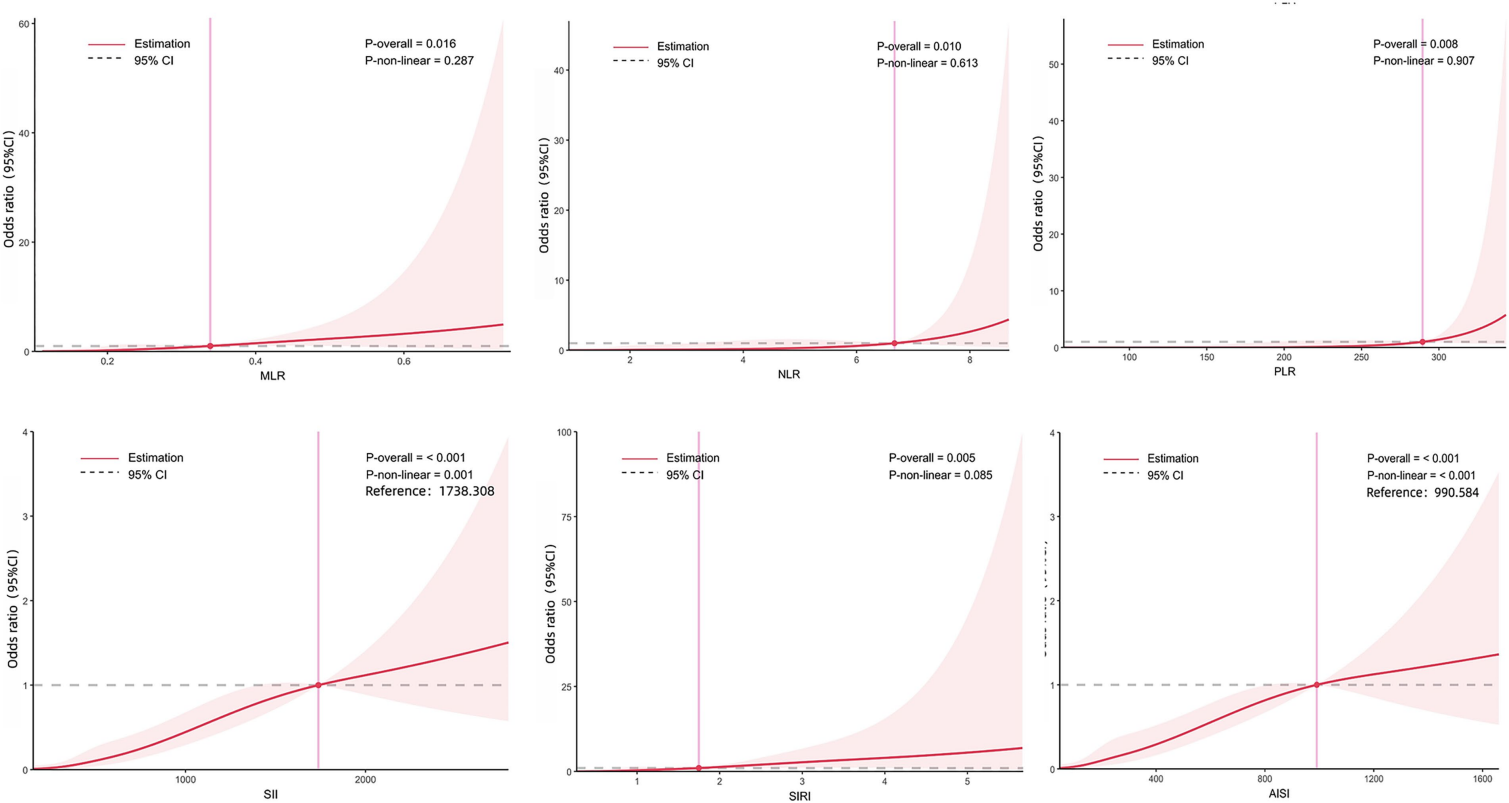
The dose–response relationship between inflammatory markers and AGA. Graphs show OR for AGA adjusted for age, TG, LDL-C, SCr, ALT, drinking, hypertension, kidney stone and fatty liver. Data were fitted by multivariate logistic regression models. Solid lines indicate OR, and shadow shapes indicate 95% CIs.

Figure 3 illustrates the dose–response associations between various inflammatory markers and AGA, using RCS for visualization and analysis. After adjusting for multiple covariates, higher inflammatory indices were associated with an increased risk of AGA. There are significant nonlinear relationships between SII and AGA (*p*-nonlinear = 0.001) as well as between AISI and AGA (*p*-nonlinear <0.001). The inflection points of the dose–response curves were identified at 1738.308 for the SII-AGA association and 990.584 for the AISI-AGA association. In contrast, MLR, NLR, PLR, and SIRI exhibited linear associations with AGA risk, as no significant nonlinearity was observed (*p*-nonlinear = 0.287, 0.613, 0.907, and 0.085, respectively).

### Subgroup analysis

**Figure 4:**
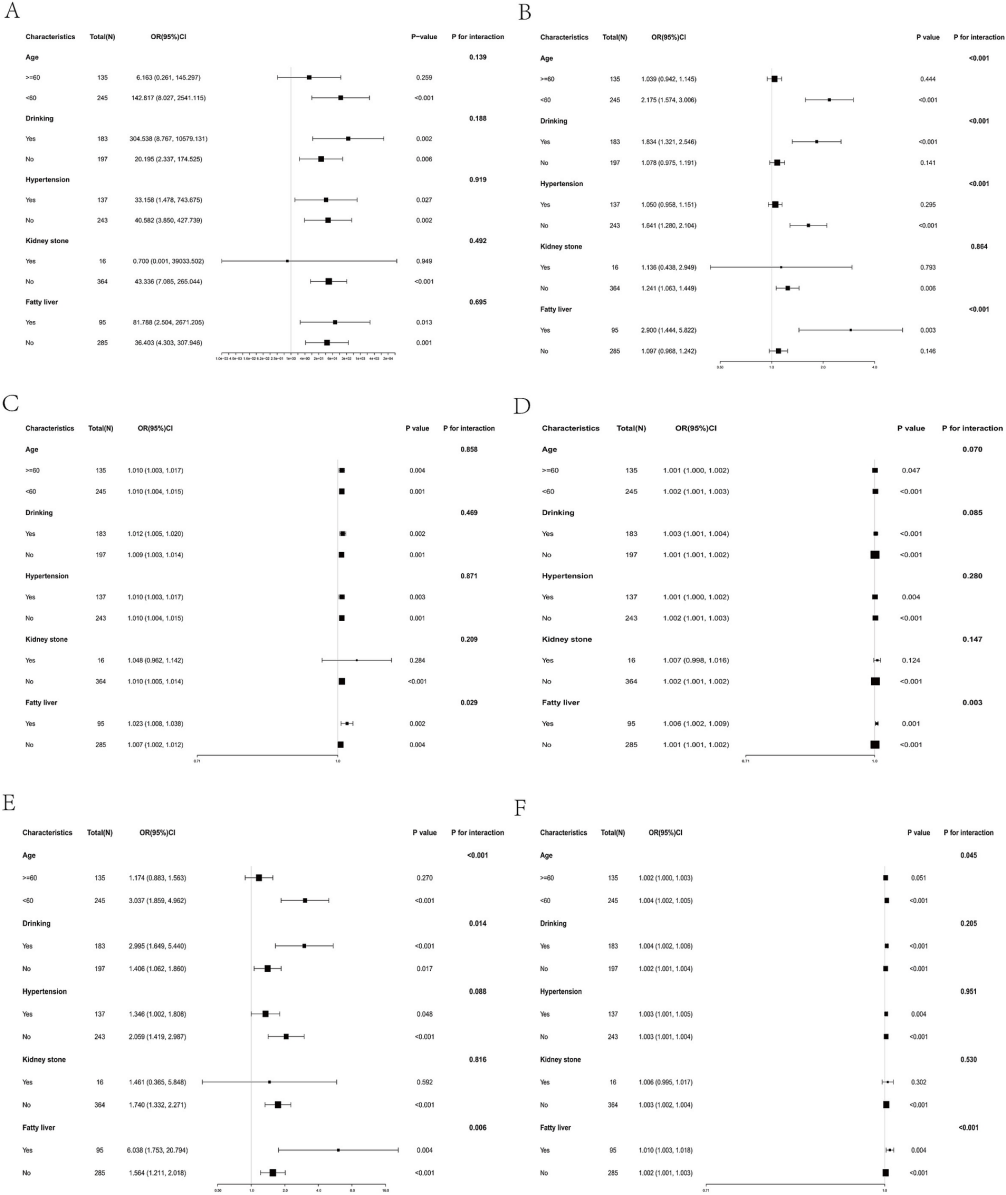
Subgroup analysis of the association between inflammatory markers and AGA. Subgroup analysis of the association between MLR **(A)**, NLR **(B)**, PLR **(C)**, SII **(D)**, SIRI **(E)** and AISI **(F)**, stratified by age, drinking, hypertension, kidney stone, and fatty liver. MLR, monocyte-to-lymphocyte ratio; NLR, neutrophil-to-lymphocyte ratio; PLR, platelet-to-lymphocyte ratio; SII, Systemic Immune-Inflammation Index; SIRI, systemic inflammatory response index; AISI, aggregate index of systemic inflammation; OR, odds ratio.

To explore the relationships of inflammatory markers with AGA in distinct subpopulations, we stratified the data by age, drinking, hypertension, kidney stone, and fatty liver. As shown in Figure 4, NLR, SIRI, and AISI exhibited significant interactions with age subgroups (*p*-interaction <0.05), with more pronounced ORs for AGA observed in individuals under 60 years of age. NLR and SIRI also demonstrated interactions with drinking status subgroups (*p*-interaction <0.05), where stronger ORs for AGA were evident in the drinking population. Notably, NLR showed a significant interaction with hypertension subgroups (*p*-interaction <0.05), with more substantial ORs for AGA observed in the non-hypertensive population. Additionally, NLR, PLR, SII, SIRI, and AISI were all associated with significant interactions with fatty liver subgroups (*p*-interaction <0.05), with more substantial ORs for AGA observed in the fatty liver population. In contrast, MLR did not exhibit interactions with any subgroups (all *p*-interaction >0.05). Given the limited sample size in the kidney stone and fatty liver subgroups, the stability of the interaction effects was further assessed using non-parametric bootstrap resampling with 5,000 iterations. In the kidney stone subgroup, none of the inflammatory markers (MLR, NLR, PLR, SII, SIRI, or AISI) showed a statistically significant interaction with disease status, as all bootstrap-derived 95% confidence intervals included zero. Conversely, in the fatty liver subgroup, significant interactions were observed for NLR, PLR, SII, SIRI, and AISI. The bootstrap 95% CIs for their respective interaction terms with fatty liver status were all entirely above zero.

### Validation of AGA diagnoses using ROC curves analysis

**Figure 5:**
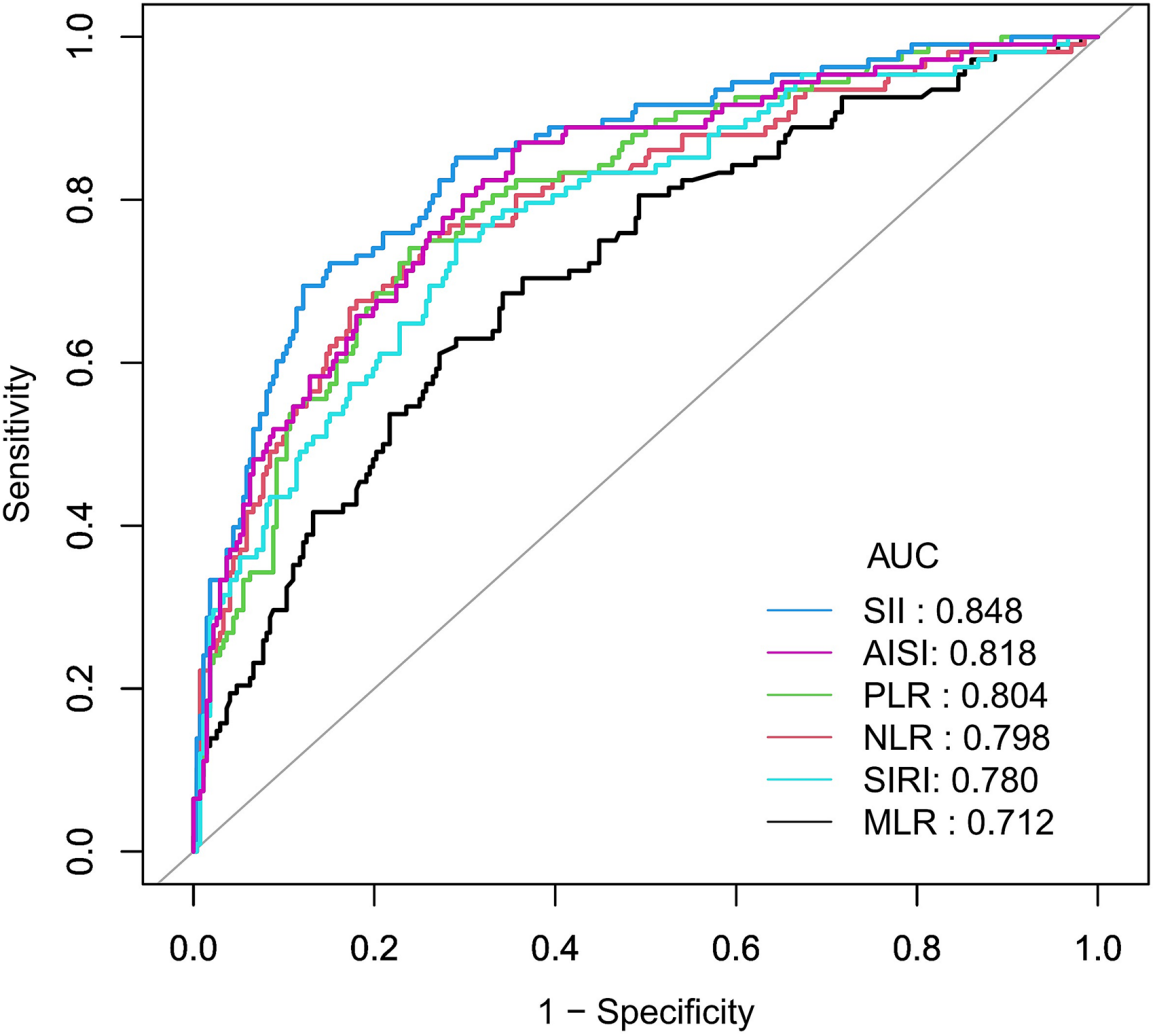
ROC curves and the AUC values of the six inflammatory markers (NLR, MLR, PLR, SII, SIRI, and AISI) in diagnosing AGA. MLR, monocyte-to-lymphocyte ratio; NLR, neutrophil-to-lymphocyte ratio; PLR, platelet-to-lymphocyte ratio; SII, Systemic Immune-Inflammation Index; SIRI, systemic inflammatory response index; AISI, aggregate index of systemic inflammation.

ROC curve analysis showed that the areas under the ROC curve (AUCs) for diagnosing AGA using MLR, NLR, PLR, SII, SIRI, and AISI were 0.712, 0.798, 0.804, 0.848, 0.780, and 0.818, respectively (Table 3; Figure 5). Among all inflammatory markers, SII and AISI had the largest AUCs for AGA diagnosis. The DeLong test further revealed that the AUC of SII was significantly higher than that of MLR, NLR, PLR, and SIRI (*Z* = 4.920, 3.539, 2.589, 3.533; all *p* < 0.05). Notably, no significant difference in AUCs was observed between SII and AISI (*p* > 0.05; Table 3). Collectively, these results suggest that SII and AISI may be more accurate and discriminative than other inflammatory markers (MLR, NLR, PLR, and SIRI) for assessing AGA risk.

**Table 3:** Performance of MLR, NLR, PLR, SII, SIRI, and AISI to evaluate AGA and optimal critical points.

Variable	AUC	95% CI	Sensitivity	Specificity	Youden index	Best threshold	*Z* statistic	*p*-value
SII	0.848	0.804–0.892	0.694	0.879	0.573	717.001	Reference	Reference
AISI	0.818	0.770–0.866	0.870	0.640	0.510	210.424	1.918	0.055
PLR	0.804	0.756–0.853	0.741	0.761	0.502	136.934	2.589	0.010
NLR	0.798	0.745–0.850	0.676	0.820	0.496	2.654	3.539	<0.001
SIRI	0.780	0.727–0.832	0.750	0.710	0.460	1.088	3.533	<0.001
MLR	0.712	0.654–0.770	0.685	0.658	0.343	0.251	4.920	<0.001

MLR, monocyte-to-lymphocyte ratio; NLR, neutrophil-to-lymphocyte ratio; PLR, platelet-to-lymphocyte ratio; SII, Systemic Immune-Inflammation Index; SIRI, systemic inflammatory response index; AISI, aggregate index of systemic inflammation; AUC, area under the curve; 95% CI, 95% confidence interval.

## Discussion

A cross-sectional study involving 380 males was conducted to investigate the association between complete blood cell count-derived inflammatory markers and the prevalence risk of AGA. The findings revealed that the MLR, NLR, PLR, SII, SIRI, and AISI were significantly associated with AGA in males. Notably, these associations persisted even after adjustment for key metabolic confounders. In individuals with fatty liver, the associations between inflammatory indices (except MLR) and AGA were notably stronger. Additionally, we employed RCS analysis to systematically explore the non-linear relationships between six selected inflammatory markers and the risks of AGA. The analysis revealed significant non-linear associations between the SII and AGA, and between the AISI and AGA. Specifically, the threshold effect analysis determined breakpoints at 1,738.308 and 990.584, respectively, indicating pivotal inflection points in these relationships. ROC analysis indicated that SII and AISI exhibited stronger discriminatory power and higher diagnostic accuracy in evaluating AGA risk. Overall, this study highlights the importance of considering inflammatory status reflected by complete blood cell count-derived markers as an independent risk factor in the population with AGA.

This study is the first to assess the relationship between NLR, MLR, PLR, SII, SIRI, and AISI with AGA risk. One of our major findings aligns with previous research. Kadiyoran et al. (13) enrolled 110 gout patients and 90 healthy volunteers as controls, collecting blood samples during the intercritical and attack phases of the patients. The study aimed to investigate whether well-known inflammation markers—including MLR, NLR, PLR, red cell distribution width (RDW), and mean platelet volume (MPV)—could serve as predictive markers for gout attacks. Findings revealed that during the attack period, NLR (*p* < 0.001), PLR (*p* < 0.05), MLR (*p* < 0.001), RDW (*p* < 0.05), and MPV (*p* < 0.05) values were significantly higher than those during the intercritical period. Regression analysis showed a strong independent association between gout attacks and MLR (*β* = 0.126, *p* < 0.001), RDW (*β* = 0.100, *p* = 0.003), and NLR (*β* = 0.082, *p* = 0.014). Thus, MLR, RDW, and NLR may serve as robust predictive markers for gout attacks. A monocentric (14), retrospective study investigated the correlation of the NLR, PLR, and MPV with gout activity. The results showed that NLR was more valuable in assessing gout disease activity. A study investigated the value of the SII and SIRI in gouty arthritis, which enrolled 474 patients with AGA, 399 patients with intercritical gouty arthritis, and 194 healthy controls. The MLR, NLR, SII, and SIRI in the AGA group were significantly higher than those in the intercritical gouty arthritis and control groups (*p* < 0.05). SIRI was an independent risk factor for acute gout attack (*p* < 0.05) (10).

We investigated the possible mechanism underlying the relationship between numerous inflammatory indices, including the NLR, PLR, MLR, SII, SIRI, and AISI, and AGA. Neutrophils are pivotal in the development of gouty arthritis (15). The core process of an acute gout attack is that sodium urate activates the NLRP3 inflammatory complex and releases bioactive IL-1β (16), which attracts a large number of neutrophils to the sodium urate site and induces a cascade of secondary inflammatory mediators such as prostaglandins and cytokines to induce an acute inflammatory response, causing pain and tissue damage (17). PLTs are indispensable for hemostasis and the induction of tissue repair. Previous studies have shown that activated PLTs play an important role in the regulation of immunity and inflammation (18, 19). Recent studies have shown that PLTs interact with different types of white blood cells during inflammation (20). When exposed to certain infections or inflammatory stimuli, white blood cells recognize platelet P-selectin or CD40 through leukocyte P-selectin glycoprotein ligand 1 and CD154, thereby forming platelet–leukocyte aggregations (21) that participate in inflammatory responses. The high abundance of NEUs promotes an increase in blood viscosity and hypercoagulability by mediating interactions between PLTs and the endothelium (22). PLTs adhere to endothelial cells, enhancing inflammation by promoting leukocyte migration and adhesion to the site of injury. Cytokines and reactive oxygen species facilitate the release of immature and activated PLTs into the peripheral blood (23). LYMs are also an important cell type involved in immune defense (24). An increase in NEUs is often accompanied by lymphopenia (25). In light of this, these indices based on LYM, NEU, and PLT counts provide a comprehensive assessment of various aspects of the body’s immune response to inflammation, enabling a more accurate prediction of disease severity (26–30). They also offer a more comprehensive and robust evaluation of the immune response related to AGA and provide novel clinical evidence for the diagnosis and prognosis of AGA. Specifically, their superior discriminatory ability enables precise differentiation between individuals at high and low risk of AGA, addressing the clinical need for rapid and reliable risk stratification in routine practice. Unlike specialized or invasive diagnostic tools, SII and AISI are derived from routine laboratory parameters that are readily accessible in primary care and emergency settings, eliminating the need for additional testing costs or procedural delays. This practical advantage not only enhances the feasibility of early AGA risk screening but also supports timely initiation of targeted interventions for high-risk populations. Furthermore, the robust diagnostic performance of these indices underscores their potential as complementary tools to conventional clinical assessment, aiding clinicians in optimizing treatment and improving long-term disease outcomes by reducing the incidence of unexpected acute flares.

In our study, we comprehensively evaluated a panel of six blood-derived inflammatory markers and subsequently identified SII and AISI as the most valuable evaluators for AGA. SII and AISI are biomarkers that incorporate multiple peripheral inflammatory components in the body, such as NEUs, LYMs, PLTs, MONOs, and other parameters related to the inflammatory response. These composite indices offer a more comprehensive evaluation of systemic inflammatory activity (31). Notably, we observed a nonlinear association between the SII, AISI, and AGA. The breakpoint of the threshold effect was calculated to be 1738.308 and 990.584, respectively. Specifically, when SII was below 1738.308 or AISI was below 990.584, the risk of AGA increased rapidly; conversely, when SII exceeded 1738.308 or AISI exceeded 990.584, the risk of AGA increased slowly. Based on the inflection point, patient risk can be accurately stratified. For patients with an SII below 1738.308 or an AISI below 990.584, implementing active anti-inflammatory treatment can significantly reduce disease risk by further decreasing AISI. However, for patients with an SII exceeding 1738.308 or an AISI exceeding 990.584, reducing AISI alone may not effectively lower disease risk. In clinical practice, more emphasis should be placed on comprehensive treatment strategies, such as using urate-lowering drugs to strictly control serum uric acid levels and rationally administering anti-inflammatory drugs to prevent acute attacks.

In the subgroup analysis, we found that the association between inflammatory markers and AGA was significantly stronger in the fatty liver subgroup than in the non-fatty liver subgroup, with a statistically significant difference. This finding can potentially be explained by the synergistic interaction between the inflammatory state and metabolic disorders associated with fatty liver. First, a study has demonstrated that non - alcoholic fatty liver disease (NAFLD) significantly elevates the risk of incident hyperuricemia (32). In cellular and murine models of NAFLD, the expression and activity of xanthine oxidase (XO), a rate-limiting enzyme in uric acid biosynthesis, were significantly upregulated. Silencing XO expression or inhibiting its activity not only significantly decreased uric acid production but also alleviated high-fat diet—induced hepatic steatosis in mice. Subsequent experiments revealed that XO regulated the activation of the NLRP3 inflammasome. NAFLD activates the NLRP3 inflammasome, thereby establishing a self-perpetuating “metabolism-inflammation” cycle and increasing the risk of AGA. Second, fatty liver is closely associated with insulin resistance, and several studies have characterized fatty liver as a hepatic manifestation of insulin resistance (33, 34). Insulin resistance impairs renal uric acid excretion in the proximal tubules, leading to hyperuricemia (35). Given that hyperuricemia is the key pathological substrate of AGA, it directly increases the risk of AGA.

This research demonstrates several notable strengths while concurrently acknowledging specific limitations. Firstly, the utilization of a geographically homogeneous sample of AGA patients from Dalian, China, effectively mitigates sampling bias and enhances the representativeness of the study population. Secondly, through the application of multivariate logistic regression analysis, stratified analyses, and interaction testing, various confounding factors were systematically controlled, thereby substantially reinforcing the reliability of the study’s findings. Moreover, the study incorporated adjustments for major confounding variables that could potentially impact the results, such as personal lifestyle habits and medical history. Additionally, inflammatory markers derived from complete blood cell count parameters, which encompass multiple indicators, have the potential to provide more comprehensive insights compared to single indices. As such, they represent valuable clinical tools for the management and treatment of adult AGA.

Notwithstanding these strengths, several limitations should be noted. First, the study did not consider AGA risk factors, including dietary patterns and medication use, which restricts the comprehensive examination of these potential confounders. Second, the medical history data, such as hypertension, diabetes, and coronary heart disease, were based on patient self-reporting. This approach may introduce bias and undermine the validity of the study results. Third, in contrast to cohort studies, the cross-sectional design employed in this research limits the exploration of causal relationships and prognostic factors. Consequently, further investigations with detailed clinical data and additional cohort studies are warranted to address these limitations. Additionally, our control group was drawn from a hospital check-up cohort, which may limit generalizability to the general population. Although this reflects a clinically relevant comparison group, future community-based studies are needed for broader validation. Furthermore, some subgroup analyses involved small sample sizes. Although bootstrap techniques were employed to enhance the robustness of associated interaction estimates, these findings should be considered exploratory and require validation in larger cohorts. Finally, the participants were diagnosed with AGA based on the 2015 ACR/EULAR gout classification criteria. Synovial fluid aspiration for MSU crystal detection was not performed due to clinical feasibility considerations. Although the 2015 ACR/EULAR classification criteria are widely recognized, potential misdiagnosis cannot be completely excluded.

## Conclusion

This study demonstrates a significant association between systemic inflammatory markers (MLR, NLR, PLR, SIRI, SII, and AISI) and the occurrence of AGA in males. Among them, SII and AISI showed the best discriminatory power for assessing AGA risk. These findings strengthen the evidence supporting the valuable role of these biomarkers in the evaluation of AGA. They have the potential to guide clinical decision-making in the comprehensive management of AGA patients, thereby facilitating personalized treatment strategies.

## Data Availability

The original contributions presented in the study are included in the article/supplementary material, further inquiries can be directed to the corresponding author.

## References

[1] Dehlin M Jacobsson L Roddy E. Global epidemiology of gout: prevalence, incidence, treatment patterns and risk factors. Nat Rev Rheumatol. (2020) 16:380–90. doi: 10.1038/s41584-020-0441-1 32541923

[2] Dalbeth N Gosling AL Gaffo A Abhishek A. Gout. Lancet. (2021) 397:1843–55. doi: 10.1016/S0140-6736(21)00569-9 33798500

[3] Taylor WJ Fransen J Jansen TL Dalbeth N Schumacher HR Brown M . Study for updated gout classification criteria: identification of features to classify gout. Arthritis Care Res (Hoboken). (2015) 67:1304–15. doi: 10.1002/acr.22585 25777045 PMC4573373

[4] Wu M Tian Y Wang Q Guo C. Gout: a disease involved with complicated immunoinflammatory responses: a narrative review. Clin Rheumatol. (2020) 39:2849–59. doi: 10.1007/s10067-020-05090-8 32382830

[5] Qin B Ma N Tang Q Wei T Yang M Fu H . Neutrophil to lymphocyte ratio (NLR) and platelet to lymphocyte ratio (PLR) were useful markers in assessment of inflammatory response and disease activity in SLE patients. Mod Rheumatol. (2016) 26:372–6. doi: 10.3109/14397595.2015.1091136, 26403379

[6] Zinellu A Mangoni AA. Neutrophil-to-lymphocyte and platelet-to-lymphocyte ratio and disease activity in rheumatoid arthritis: a systematic review and meta-analysis. Eur J Clin Investig. (2023) 53:e13877. doi: 10.1111/eci.13877 36121342

[7] Al-Osami MH Awadh NI Khalid KB Awadh AI. Neutrophil/lymphocyte and platelet/lymphocyte ratios as potential markers of disease activity in patients with ankylosing spondylitis: a case-control study. Adv Rheumatol. (2020) 60:13. doi: 10.1186/s42358-020-0113-5 32000859

[8] Xu Y He H Zang Y Yu Z Hu H Cui J . Systemic inflammation response index (SIRI) as a novel biomarker in patients with rheumatoid arthritis: a multi-center retrospective study. Clin Rheumatol. (2022) 41:1989–2000. doi: 10.1007/s10067-022-06122-1 35266094

[9] Zengin O Göre B Öztürk O Cengiz AM Güler Kadıoğlu S Asfuroğlu Kalkan E . Evaluation of acute pancreatitis severity and prognosis using the aggregate systemic inflammation index (AISI) as a new marker: a comparison with other inflammatory indices. J Clin Med. (2025) 14:3419. doi: 10.3390/jcm14103419 40429414 PMC12111921

[10] Jiang Y Tu X Liao X He Y Wang S Zhang Q . New inflammatory marker associated with disease activity in gouty arthritis: the systemic inflammatory response index. J Inflamm Res. (2023) 16:5565–73. doi: 10.2147/JIR.S432898, 38034046 PMC10683657

[11] GBD 2021 Gout Collaborators. Global, regional, and national burden of gout, 1990–2020, and projections to 2050: a systematic analysis of the Global Burden of Disease Study 2021. Lancet Rheumatol. (2024) 6:e507–17. doi: 10.1016/S2665-9913(24)00117-6 38996590 PMC11263476

[12] Neogi T Jansen TLTA Dalbeth N Fransen J Schumacher HR Berendsen D . 2015 gout classification criteria: an American College of Rheumatology/European league against rheumatism collaborative initiative. Arthritis Rheumatol. (2015) 67:2557–68. doi: 10.1002/art.39254 26352873 PMC4566153

[13] Kadiyoran C Zengin O Cizmecioglu HA Tufan A Kucuksahin O Cure MC . Monocyte to lymphocyte ratio, neutrophil to lymphocyte ratio, and red cell distribution width are the associates with gouty arthritis. Acta Med (Hradec Kralove). (2019) 62:99–104. doi: 10.14712/18059694.2019.132 31663502

[14] Wu H Zhou H Chen P. Correlation of neutrophil-lymphocyte ratio (NLR), platelet-lymphocyte ratio (PLR), and mean platelet volume (MPV) with gout activity: a monocentric and retrospective study. Medicine (Baltimore). (2022) 101:e30242. doi: 10.1097/MD.0000000000030242 36107534 PMC9439824

[15] Mitroulis I Kambas K Chrysanthopoulou A Skendros P Apostolidou E Kourtzelis I . Neutrophil extracellular trap formation is associated with IL-1β and autophagy-related signaling in gout. PLoS One. (2011) 6:e29318. doi: 10.1371/journal.pone.0029318, 22195044 PMC3241704

[16] Wang J Zhou D Dai Z Li X. Association between systemic immune-inflammation index and diabetic depression. Clin Interv Aging. (2021) 16:97–105. doi: 10.2147/CIA.S285000, 33469277 PMC7810592

[17] Tong Y-S Tan J Zhou X-L Song Y-Q Song Y-J. Systemic immune-inflammation index predicting chemoradiation resistance and poor outcome in patients with stage III non-small cell lung cancer. J Transl Med. (2017) 15:221. doi: 10.1186/s12967-017-1326-1 29089030 PMC5664920

[18] Thomas MR Storey RF. The role of platelets in inflammation. Thromb Haemost. (2015) 114:449–58. doi: 10.1160/TH14-12-1067 26293514

[19] Mandel J Casari M Stepanyan M Martyanov A Deppermann C. Beyond hemostasis: platelet innate immune interactions and thromboinflammation. Int J Mol Sci. (2022) 23:3868. doi: 10.3390/ijms23073868, 35409226 PMC8998935

[20] Klinger MHF Jelkmann W. Role of blood platelets in infection and inflammation. J Interf Cytokine Res. (2002) 22:913–22. doi: 10.1089/10799900260286623, 12396713

[21] Zhang F Niu M Wang L Liu Y Shi L Cao J . Corrigendum: systemic-immune-inflammation index as a promising biomarker for predicting perioperative ischemic stroke in older patients who underwent non-cardiac surgery. Front Aging Neurosci. (2022) 14:1101574. doi: 10.3389/fnagi.2022.1101574, 36570527 PMC9773973

[22] Li X Ji Y Kang J Fang N. Association between blood neutrophil-to-lymphocyte ratio and severity of coronary artery disease: evidence from 17 observational studies involving 7017 cases. Medicine (Baltimore). (2018) 97:e12432. doi: 10.1097/MD.0000000000012432 30278521 PMC6181556

[23] Cure MC Cure E Kirbas A Cicek AC Yuce S. The effects of Gilbert’s syndrome on the mean platelet volume and other hematological parameters. Blood Coagul Fibrinolysis. (2013) 24:484–8. doi: 10.1097/MBC.0b013e32835e4230, 23348429

[24] de Lima JD de Paula AGP Yuasa BS de Souza Smanioto CC da Cruz Silva MC Dos Santos PI . Genetic and epigenetic regulation of the innate immune response to gout. Immunol Investig. (2023) 52:364–97. doi: 10.1080/08820139.2023.2168554 36745138

[25] Zahorec R. Ratio of neutrophil to lymphocyte counts—rapid and simple parameter of systemic inflammation and stress in critically ill. Bratisl Lek Listy. (2001) 102:5–14. 11723675

[26] Tarle M Raguž M Lukšić I. A comparative study of the aggregate index of systemic inflammation (AISI) and C-reactive protein (CRP) in predicting odontogenic abscesses severity: a novel approach to assessing immunoinflammatory response. Diagnostics (Basel). (2024) 14:2163. doi: 10.3390/diagnostics14192163 39410567 PMC11475933

[27] Mangoni AA Zinellu A. The diagnostic role of the systemic inflammation index in patients with immunological diseases: a systematic review and meta-analysis. Clin Exp Med. (2024) 24:27. doi: 10.1007/s10238-024-01294-3 38285324 PMC10824868

[28] Buonacera A Stancanelli B Colaci M Malatino L. Neutrophil to lymphocyte ratio: an emerging marker of the relationships between the immune system and diseases. Int J Mol Sci. (2022) 23:3636. doi: 10.3390/ijms23073636, 35408994 PMC8998851

[29] Wang G Mivefroshan A Yaghoobpoor S Khanzadeh S Siri G Rahmani F . Prognostic value of platelet to lymphocyte ratio in sepsis: a systematic review and meta-analysis. Biomed Res Int. (2022) 2022:9056363. doi: 10.1155/2022/9056363 35707370 PMC9192240

[30] Nishijima TF Muss HB Shachar SS Tamura K Takamatsu Y. Prognostic value of lymphocyte-to-monocyte ratio in patients with solid tumors: a systematic review and meta-analysis. Cancer Treat Rev. (2015) 41:971–8. doi: 10.1016/j.ctrv.2015.10.003 26481060

[31] Cao C Li C Li X Sun W Wang Y. Association of systemic immune-inflammation index (SII) and aggregate index of systemic inflammation (AISI) with thyroid nodules in patients with type 2 diabetes mellitus: a retrospective study. BMC Endocr Disord. (2023) 23:251. doi: 10.1186/s12902-023-01509-w 37986076 PMC10659038

[32] Xu C Wan X Xu L Weng H Yan M Miao M . Xanthine oxidase in non-alcoholic fatty liver disease and hyperuricemia: one stone hits two birds. J Hepatol. (2015) 62:1412–9. doi: 10.1016/j.jhep.2015.01.019, 25623823

[33] Itabashi F Hirata T Kogure M Narita A Tsuchiya N Nakamura T . Combined associations of liver enzymes and obesity with diabetes mellitus prevalence: the Tohoku medical megabank community-based cohort study. J Epidemiol. (2022) 32:221–7. doi: 10.2188/jea.JE20200384 33390464 PMC8979920

[34] Choi A Kim JH Chung H-K Ahn CW Choi HJ Kim Y-S . The effects of *C. lacerata* on insulin resistance in type 2 diabetes patients. J Diabetes Res. (2022) 2022:1–10. doi: 10.1155/2022/9537741, 35242882 PMC8888035

[35] Asma Sakalli A Küçükerdem HS Aygün O. What is the relationship between serum uric acid level and insulin resistance?: a case-control study. Medicine (Baltimore). (2023) 102:e36732. doi: 10.1097/MD.0000000000036732, 38206747 PMC10754590

